# Microstructure Influence of SACX0307-TiO_2_ Composite Solder Joints on Thermal Properties of Power LED Assemblies

**DOI:** 10.3390/ma13071563

**Published:** 2020-03-28

**Authors:** Agata Skwarek, Przemysław Ptak, Krzysztof Górecki, Tamás Hurtony, Balázs Illés

**Affiliations:** 1 Department of Marine Electronics, Gdynia Maritime University, 81-255 Gdynia, Poland; p.ptak@we.umg.edu.pl (P.P.); k.gorecki@we.umg.edu.pl (K.G.); 2 Department of Microelectronics, Łukasiewicz Research Network–Institute of Electron Technology, 30-701 Kraków, Poland; 3 Department of Electronics Technology, Budapest University of Technology and Economics, 1111 Budapest, Hungary; hurtony@ett.bme.hu (T.H.); billes@ett.bme.hu (B.I.)

**Keywords:** TiO_2_ ceramic, SAC composite alloy, microstructure characterization, thermal resistance, luminous efficiency, power LED

## Abstract

The effect of the microstructure of solder joints on the thermal properties of power LEDs is investigated. Solder joints were prepared with different solder pastes, namely 99Sn0.3Ag0.7Cu (as reference solder) and reinforced 99Sn0.3Ag0.7Cu–TiO_2_ (composite solder). TiO_2_ ceramic was used at 1 wt.% and with two different primary particle sizes, which were 20 nm (nano) and 200 nm (submicron). The thermal resistance, the electric thermal resistance, and the luminous efficiency of the power LED assemblies were measured. Furthermore, the microstructure of the different solder joints was analyzed on the basis of cross-sections using scanning electron and optical microscopy. It was found that the addition of submicron TiO_2_ decreased the thermal and electric thermal resistances of the light sources by 20% and 16%, respectively, and it slightly increased the luminous efficiency. Microstructural evaluations showed that the TiO_2_ particles were incorporated at the Sn grain boundaries and at the interface of the intermetallic layer and the solder bulk. This caused considerable refinement of the Sn grain structure. The precipitated TiO_2_ particles at the bottom of the solder joint changed the thermodynamics of Cu_6_Sn_5_ formation and enhanced the spalling of intermetallic grain to solder bulk, which resulted in a general decrease in the thickness of the intermetallic layer. These phenomena improved the heat paths in the composite solder joints, and resulted in better thermal and electrical properties of power LED assemblies. However, the TiO_2_ nanoparticles could also cause considerable local IMC (Intermetallic Compounds) growth, which could inhibit thermal and electrical improvements.

## 1. Introduction

Solid-state light sources become prevailing devices in the lighting technique [1,2,3]. An essential component of them is the power LED (Light Emitting Diode) [1,2,3,4], in which the conversion of electrical energy into light takes place. During this conversion, some energy is changed into heat and increases the internal temperature of the diodes as a self-heating phenomenon [4,5,6,7,8]. The increase in internal temperature decreases the emitted luminous flux and worsens the luminous efficiency (*η_Ε_*) of solid-state light sources [8,9]. At very high temperatures (e.g., 120 °C), the *η_Ε_* can decrease several times [10]. Therefore, the efficient heat management of power LEDs is crucial [4,10,11]. The efficiency of heat removal from the LED can be described using the thermal resistance of the power LED assembly:

(1) $$R_{th}=\frac{T_{j}-T_{a}}{P_{th}}$$

where *T_a_* is the ambient temperature (K), *T_j_* is the internal temperature (K), and *P_th_* is the dissipated thermal power of the semiconductor device [12,13]. The *R_th_* is determined by the elements of the device through which the heat flows, e.g., the die of the chip, the package of the component, the substrate of the assembly, the heat-sink, and the case of the device [14]. The thermal proprieties of the connecting elements between these different parts are also important, such as the thermal interfaces and solder joints [15]. Other important factors that influence the efficiency of heat dissipation from electronic components are the soldering method, circuit layout, and solder alloy composition [16,17]. Previous studies have proved the influence of the assembly process, and the thermal pad dimension of Schottky diodes’ die on the thermal resistance of the diodes [13,18]. The influence of the soldering method, related to the void ratio in the solder joint, on the thermal resistance of the power LED was also investigated [15]. Other thermal problems and solutions of power LEDs are reported in [19,20]. These problems are connected to the thermal modelling of power LEDs and multi-domain modelling of such semiconductor devices. However, the influence of the modification of solder joints on the thermal properties of power LEDs is still questionable.

Up to 2006, tin-lead alloys (e.g., Sn63Pb37 or Sn60Pb40) were mainly used in the electronics industry. Transition to lead-free technologies introduced the widespread use of tin-silver-copper (SnAgCu, SAC) solder alloys, e.g., Sn96.5Ag3Cu0.5 (SAC305) or Sn95.5Ag4Cu0.5 (SAC405). However, the main disadvantage of lead-free SAC alloys is their poor mechanical performance due to their high Ag content [21]. Therefore, new research is being conducted to reduce the Ag content in SAC solders below the hypereutectic composition (up to an Ag content of 1.35 wt.%), maintaining their melting point between 217–221 °C. Moreover, because of the rapid miniaturization of electronic products, the available soldering technology may not guarantee the required performance, e.g., the I/O counts of IC chips [22]. Solder alloys with better mechanical and thermal properties are required [22]. One solution is to add ceramic powders and nanopowders to the solder alloys as a reinforcement. The variety of ceramics was already applied, like TiO_2_, ZnO, Si_3_Ni_4_, SiC, La_2_O_3_, ZrO_2_, etc. [23,24,25]. Unfortunately, some of these reinforcements can be excluded from the solder joint during the soldering process [26]. The retained ratio is usually higher when the composite solder was prepared using powder metallurgy than when using a solder-paste blending method [26].

Recently, TiO_2_ is the most frequently used reinforcement material. Grain refinement is a well-established process resulting in enhanced metallurgical properties such as toughness, ductility, and yield strength [27]. The reinforcement addition could modify the surface instability [28], the variation in the physical properties, and the grain boundary/interfacial characteristics [28,29]. The solder joints of the SAC alloy are composed of β–Sn matrix and Intermetallic Compounds (IMCs), usually Cu_6_Sn_5_, Cu_3_Sn, and Ag_3_Sn [30]. TiO_2_ particles can change several parameters of the solder alloy. First of all, the addition of TiO_2_ influences the IMC growth. On the one hand, it decreases the thickness of the IMC layer [31]; on the other, it influences the size of the Ag_3_Sn grains, as well as the spacing between the Ag_3_Sn grains [27,31]. The reduction of the spacing between the Ag_3_Sn grains significantly increases the microhardness of the material [27]. TiO_2_ influence in the microstructural changes is related to the presence of enriched (with TiO_2_) regions at the grain boundaries and increased microporosity [31,32]. The microhardness of the solder joint is influenced by TiO_2_ by pinning the grain boundaries and thus impeding sliding of the grain boundaries, the increase in dislocation densities, and obstacles to restrict the motion of dislocation and the hardening mechanism of the matrix and TiO_2_ nanopowders [33,34]. An addition of TiO_2_ nanopowders has a significant effect on the ultimate tensile stress of lead-free SAC composite solders, increasing the value of this parameter of 25%. It also causes a 0.2% offset strain (0.2 YS) increase of 30%, as compared to noncomposite solder alloys. However, the total elongation of the lead-free SAC solder containing TiO_2_ nanopowders is less than that of the lead-free SAC solder [31]. Tsao et al. [31] found that the solidus (TS) and liquidus (TL) temperatures of lead-free SAC solder reinforced with 1 wt.% of TiO_2_ increased slightly from 220.1 to 220.9 °C and from 223.6 to 226.8 °C. Since TiO_2_ has a high melting point, this increase in the melting point of TiO_2_-reinforced composite solder alloys suggests the TiO_2_ particles are dissolved locally in the molten solder. Ramli et al. [28] observed decreases in the CTE value with increasing TiO_2_ wt.% because the dimensional stability of the Sn–0.7Cu–0.05Ni composite solder is better than that of the non-reinforced solder. Better thermal-fatigue reliability is recommended, so a lower value of CTE is preferable [28,35].

The effect of TiO_2_ reinforcement on the mechanical parameters of the solder joint has been widely researched. However, the influence of TiO_2_ on the thermal properties of the solder joints and the whole assembly has never been described. Therefore, the aim of this study is to describe the influence of the composite solder alloy on the thermal properties of power LED assemblies by characterizing the relationship between the microstructure of the composite solder joints and their thermal properties.

## 2. Materials and Methods

### 2.1. Preparation of Samples

**Figure 1:**
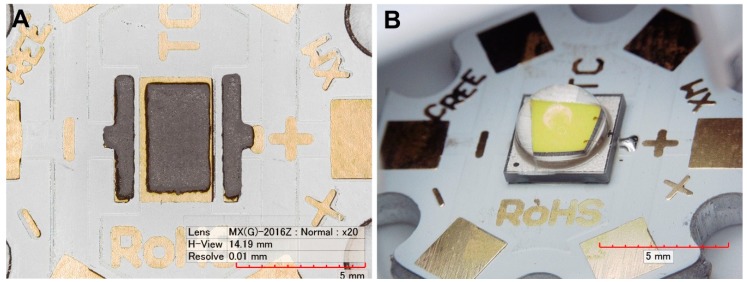
The sample circuit: (**A**) solder paste (SACX0307-nanoTiO_2_) coverage of the thermal pad; (**B**) assembled circuit.

Alumina substrates (Star MCPCB board with 22 mm diameter) 2.4 mm thick (CREE Inc., Research Triangle Park, NC, USA) were used, which were covered with a 60 μm thick Cu layer. A surface finish was prepared from the galvanic Ni/Au layer with a thickness of 2.5 µm/0.25 µm. Composite solder alloys were produced from SACX0307 (Sn99Ag0.3Cu0.7) solder paste (Alpha Industries, Chantilly, VA, USA) with the addition of 1.0 wt.% of TiO_2_ reinforcements (Sigma-Aldrich, St. Louis, MO, USA), either primary particle size 20 nm (nanoparticles), or primary particle size 200 nm (submicron particles). Both parts were mixed homogeneously using the ball milling process, which was carried out for 10 min at 300 rpm using a planetary ball mill Pulverisette 5 (Fritsch GmBh., Idar-Oberstein, Germany). As the reference sample, pure SACX0307 was used. The solder joints were prepared using stencil printing with a stencil thickness of 125 µm. To avoid a short cut during the soldering, the stencil aperture was narrowed by 10% over the thermal pad (Figure 1A). 

The samples are referred to as SACX0307, SACX0307-nanoTiO_2_, and SACX0307-TiO_2_. The samples were soldered in a convection reflow oven SMT 460C (Essemtec AG, Aesch, Switzerland) with a linear temperature profile: pre-heating (60 s, 160–180 °C), reflowing (40 s, 213–247 °C), and cooling (70 s, 247–150 °C). The convection reflow took place in an air atmosphere. 

For the experiment, XMLBWT-02-0000-000HT20E7 power LEDs (CREE Inc., Research Triangle Park, NC, USA) were used with a maximum forward current *I_Fmax_* = 3 A and a maximum admissible internal temperature *T_jmax_* = 150 °C. Thermal resistance between the junction of this LED and the soldering point is *R_thj-s_* = 2.5 K/W. The diode is characterized by its correlated color temperature CCT = 3000 K, whereas the generated luminous flux Φ*_v_* = 200 lm at the forward current *I_F_* = 700 mA and junction temperature *T_j_* = 85 °C. The assembled circuit is shown in Figure 1B. 

### 2.2. Thermal and Optical Measurements

**Figure 2:**
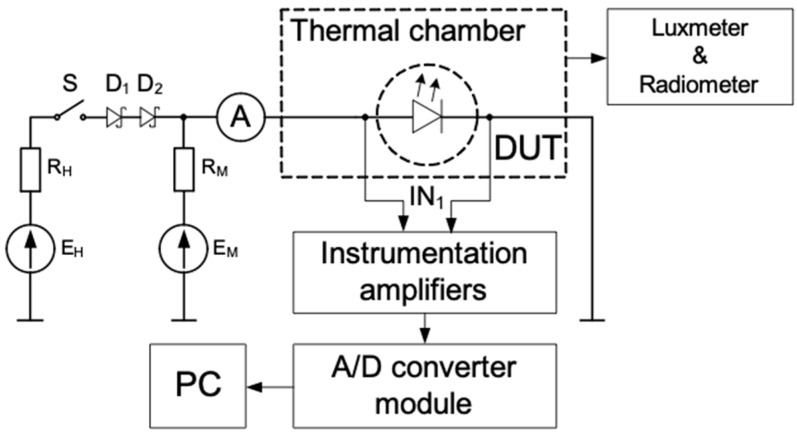
Measurement set-up scheme.

The LED test system was used to measure the DC current-voltage characteristics of the diodes, their thermal resistance, and the illuminance of the light emitted (Figure 2).

While the measurements were being taken, the samples were placed into a light-tight chamber and mounted onto a heat exchanger (liquid cooling). A luxmeter (by Sonopan, Białystok, Poland) measured the illuminance of the light emitted, and the radiometer (by Delta Ohm, Caselle, Italy) measured the power density of this light. The thermal resistance was measured using the indirect electrical method [7], which means that the voltage drop V_D_ on the diode (DUT) at a fixed value of the forward current (I_M_) was used as thermally-sensitive parameters. The voltage source (E_M_) with a resistor (R_M_) ensured the fixed value I_M_. The voltage source (E_H_) with a resistor (R_H_) produced the heating current that flowed through the diode being tested. The switch (S) was closed while these diodes were being heated, and it was open while they were being cooled. The waveforms of the diode forward voltage were recorded using an instrumentation amplifier, an A/D converter module, and a PC. The heating current was measured using an ammeter.

The thermal resistance was measured in 4 steps. In the first step, the thermometric characteristic was measured, which describes the dependence of the voltage drop (*V_D_*) on the temperature. During this step, the switch (S) was open, and I_M_ flowed through the diode. In the second step, the switch (S) was closed, and the I_H_ flowed through the diode and increased the junction temperature. This temperature rise resulted in the forward voltage (*V_H_*) change of the diode. The thermal steady-state was reached when the forward voltage did not change more than the discretization error of A/D converter for 5 min. In the steady-state, the I_H_ current, the V_H_ forward voltage, and the illuminance were measured. In the third step, the switch (S) was opened, and the forward voltage of the diode (*V_L_*) was measured at the current (*I_M_*). Finally, the value of thermal resistance was calculated using the following formula

(2) $$R_{th}=\frac{V_{L}-V_{D}\left(T_{a}\right)}{\alpha_{T}\cdot P_{th}}$$

where *α_T_* is the slope of thermometric characteristic, *P_th_* is the heating power, and *T_a_* is the ambient temperature. In Equation (2), the power *P_th_* is equal to the difference between the power received from the power supply and the radial power. The radial power was determined according to the measured power density of the light emitted, and the data-sheet of the LED being tested [14]. On the other hand, the electric thermal resistance *R_the_* was determined using the following equation:

(3) $$R_{the}=\frac{V_{L}-V_{D}\left(T_{a}\right)}{\alpha_{T}\cdot I_{H}\cdot V_{H}}$$

The luminous efficiency (*η_F_*) is the quotient of emitted luminous flux Φ*_V_* and the power of the power supply used. The luminous flux was calculated using an indirect method [10]:

(4) $$\Phi_{V}=2\pi \cdot r^{2}\cdot E\cdot (1-\cos\alpha )\cdot \alpha_{opt}$$

where *E* is the illuminance, *r* is the distance between the probe of the illuminometer and the LED being examined (r = 255 mm), α is the angle of emission (α = 85°, given by data-sheet of the LED), and α*_opt_* marks the average value of luminous intensity inside the angle of emission. While the measurements were being taken, the assembly was placed on a heat exchanger, which was an Ocool fluidic cooling system (Alphacool Company, Braunschweig, Germany). The cooling system kept the assembly at a constant room temperature (not exceeding 25 °C during measurements).

### 2.3. Microstructural Investigation of the Solder Joints

The void ratio of the solder joints was measured using XiData6600 2D X-ray (Nordson Dage, Westlake, OH, USA). The microstructure of the joints was analyzed in metallographic cross-sections. For the observations, two different Scanning Electron Microscope (SEM) were used, namely FEI Inspect S50 simple thermal emission-SEM (Thermo Fisher Scientific, Waltham, MA, USA) and Thermo Scientific Scios 2 ultra-high resolution non-immersion field emission-SEM (Thermo Fisher Scientific, Waltham, MA, USA). A Seconder Electron detector (SE) and a Back Scattered Electron (BSE) detectors were used. Energy-Dispersive X-ray spectroscopy (EDX) analysis was used to identify the elemental composition of the samples. Surface cuts were prepared on the cross-sections using Thermo Scientific Scios 2 Focused Ion Beam FIB (Thermo Fisher Scientific, Waltham, MA, USA) to investigate the incorporation of the ceramic particles into the solder matrix. 

## 3. Results and Discussion

Electric, thermal, and optical parameters of power LEDs were measured at the power supply of the diodes used as a direct current. The maximum admissible forward current of the diodes (*I_Fmax_* = 3 A) was increased to 5 A with the forced cooling system. It resulted in a higher junction temperature of the LEDs over the ambient temperature, and it allowed for more accurate measurement of *R_th_* and *R_the_*. The measurement error of the thermal resistance is diminished due to the use of a high forward current, which resulted in a high temperature inside the LED [36]. This allowed subtle changes in the thermal resistances to be observed, which resulted from modifying the composition of the soldering paste. 

**Figure 3:**
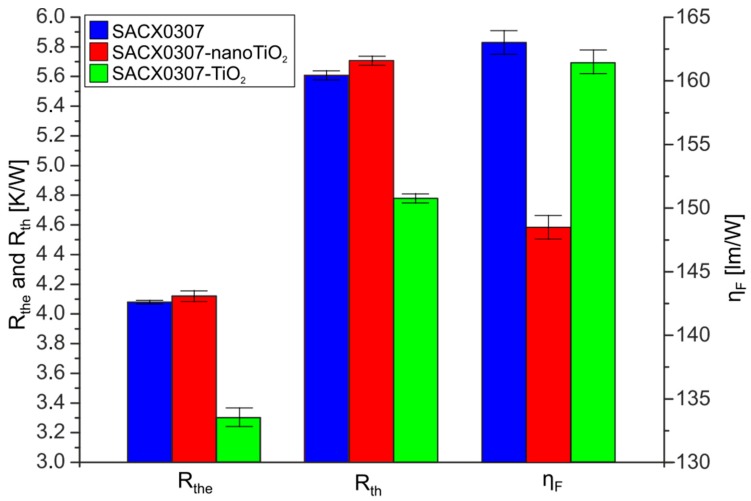
Thermal resistance (*R_th_*), electric thermal resistance (*R_the_*), and luminous efficiency (*η_F_*) of the tested diodes soldered by different soldering pastes (*I_Fmax_* = 5 A).

The values of electric thermal resistance *R_the_*, thermal resistance *R_th_*, and the luminous efficiency *η_F_* in the case of the different samples are presented in Figure 3. 

The electric thermal resistance *R_the_* of the reference sample (SACX0307) and the SACX0307-TiO_2_ sample were almost equal. These values ranged between 4.05 K/W and 4.15 K/W. These deviations between the values of *R_th_* correspond to the measurement error. However, the *R_the_* value of SAC0307-nanoTiO_2_ samples was 20% lower than those of the other samples. The decrease was 0.8 W/K. The whole electric thermal resistance between the junction of the LED and surroundings decreased by 16% as well. *R_the_* values were lower than the *R_th_* values in all cases. The difference was as high as 40%. It suggests that as much as 40% of the electrical energy received from the power source was changed into light. The efficiency of the conversion of electrical energy into the luminous flux *η*_F_ varied depending on the type of soldering paste. The improvement of the thermal properties slightly increased the luminescence efficiency of SACX0307-TiO_2_. However, the minor increase in the thermal resistance of the SACX0307-nanoTiO_2_ solder joints resulted in a 12% luminescence efficiency decrease. This suggests that the size of the reinforcement particle is significant. The worst thermal and electrical parameters (the highest thermal resistance and the lowest luminous efficiency) were observed for the samples soldered with SACX0307-nanoTiO_2_ and the best for the samples soldered with SACX0307-TiO_2_.

**Figure 4:**
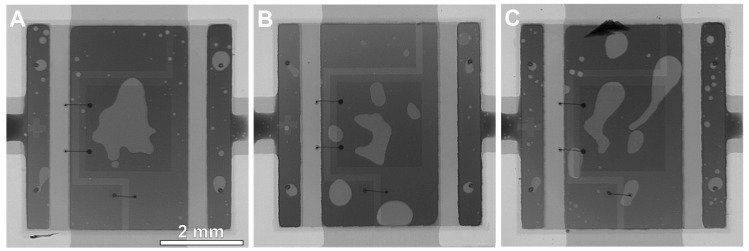
X-ray images of the solder joints under the LED component: (**A**) reference SACX0307 sample; (**B**) SACX0307-nanoTiO_2_ sample; (**C**) SACX0307-TiO_2_ sample.

One of the possible reasons for such changes could be the number of solder voids in the joints. The addition of TiO_2_ influences the wettability of the solder alloy [28]. As was proved by Ramli et al. [28], the wettability of the composite solder alloys is better than non-composite solder alloys. This is related to the lower surface tension of composite solder alloys, which decreases the contact angle and thereby improves the wettability. TiO_2_ can act as an agent to decrease the contact angle due to the segregation of TiO_2_ particles near the wetting triple point [28]. Lower surface tension significantly reduces the amount of solder voids [37]. However, in our research, the area ratio of solder voids (lighter gray spots in Figure 4) was similar for all of the samples.

The void content was 11–13%, which is acceptable in the case of the big component with large cooling pads. The 11–13% of void content does not influence the thermal parameters of the LED circuit.

**Figure 5:**
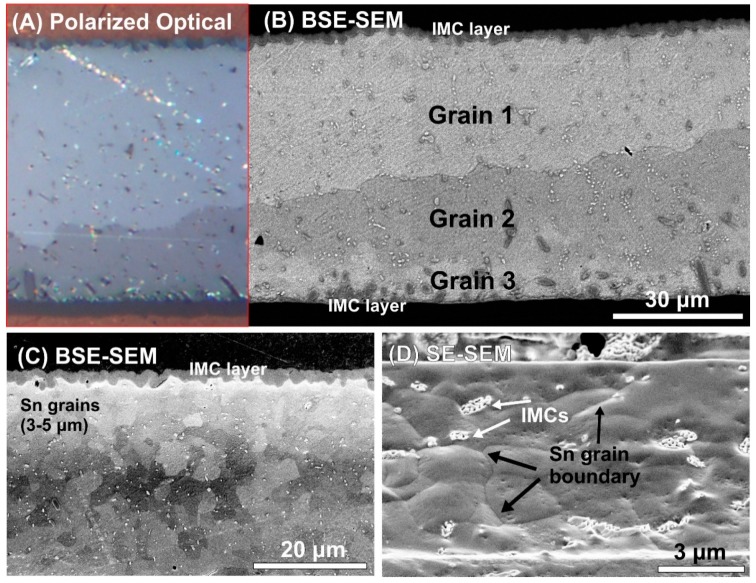
Sn grain size in the different samples: (**A**) Polarized optical image of SACX0307 sample; (**B**) BSE-SEM micrograph of SACX0307 sample; (**C**) BSE-SEM micrograph of SACX0307-TiO_2_ sample at the upper IMC layer; (**D**) tilted SE-SEM micrograph of SACX0307-nano TiO_2_ sample in the bulk solder.

The differences in thermal parameters were probably caused by the changes in the solder joint microstructure related to the presence of TiO_2_ ceramics. Figure 5 shows the microstructure of the cross-sectioned solder joints.

The use of both types of TiO_2_ ceramic particles (nano and submicron) significantly suppressed the Sn grain growth. The reference samples (SACX0307) were investigated using polarized optical imaging and BSE-SEM as well (Figure 5A,B) to localize the large Sn grains. The grain size in these solder joints was as large as hundreds of micro-meters, which is typical for simple solder alloys. The whole solder joint contained only a few grains with different orientations. On the contrary, in the case of composite solder alloys (both types SACX0307-TiO_2_, as well as SACX0307-nanoTiO_2_), the average grain size decreased to 3–5 μm, which means grain refinement of 2 orders of magnitude (Figure 5C,D). Grain size differences were not observed between composite solder alloys; this means it was equal for the samples SACX-TiO_2_ and SACX-nanoTiO_2_.

**Figure 6:**
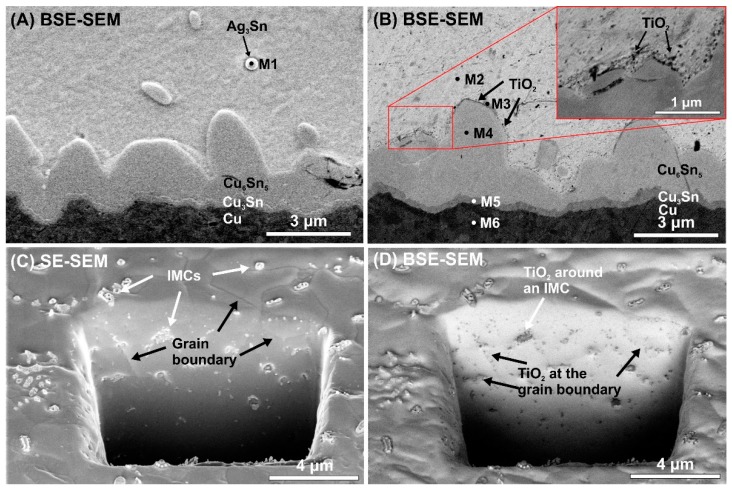
Locations of TiO_2_ nano-particles in the solder matrix: (**A**) BSE-SEM micrograph of cross-sectioned SACX0307 sample at the bottom IMC layer; (**B**) BSE-SEM micrograph of a cross-sectioned SACX0307-nanoTiO_2_ sample at the bottom IMC layer; (**C**) SE-SEM micrograph of a SACX0307-nanoTiO_2_ sample in the solder bulk; (**D**) BSE-SEM micrograph of a SACX0307-nanoTiO_2_ sample in the solder bulk.

Figure 6 presents the incorporation of the nanoTiO_2_ particles into the solder matrix, and Table 1 presents the elemental composition of the different phases in the solder joints (oxygen and carbon were excluded from the EDX results).

Figure 6A,B shows the differences between the IMC layer regions of the composite and the reference solder joints. In the composite solder joints, the TiO_2_ particles are located on the surface of the IMC layer. Figure 6C,D shows the same FIB cut in the solder bulk of a SACX0307-nanoTiO_2_ sample by SE and BSE detectors. When an SE detector is used, the IMC particles and the grain boundaries are visible. When a BSE detector’s used, the nanoTiO_2_ particles are visible. A comparison of the two micrographs (Figure 6C,D) reveals that the TiO_2_ particles are located at the grain boundaries and around the IMC particles. The TiO_2_ particles cannot be dissolved in the molten solder, so during the solidification of the solder joint, they precipitate at the grain boundaries and around the IMCs. It might cause the suppressing effect of the TiO_2_ particles on the IMC layer and Sn grain growth in the composite solder joints, discussed previously.

The thickness of the IMC layer, as well as the volume fraction of the IMC particles, are important parameters from the point of view of the electrical and thermal conductivity of the solder joints. The structure of the solder joints can be distinguished because it is rather like “a sandwich” structure, with upper and bottom parts. The upper part means the interface between the component (diode) and the solder joint, the bottom part corresponds to the interface between the solder joint and the substrate. Both interfaces contain a Cu layer, which takes part in the formation of IMC layers.

**Figure 7:**
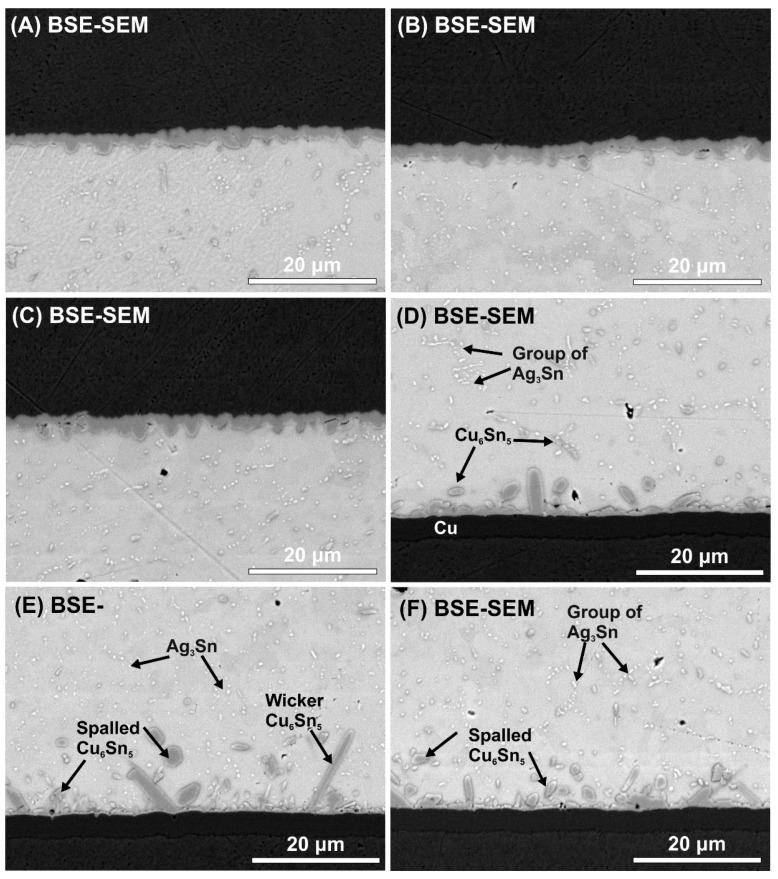
BSE-SEM micrographs of IMC layers in the different samples: (**A**) upper IMC layer in a SACX0307 sample; (**B**) upper IMC layer in a SACX0307-TiO_2_ sample; (**C**) upper IMC layer in a SACX0307-nano TiO_2_ sample; (**D**) bottom IMC layer in a SACX0307 sample; (**E**) bottom IMC layer in a SACX0307-TiO_2_ sample; (**F**) bottom IMC layer in a SACX0307-nano TiO_2_ sample.

The average thicknesses of the upper and bottom IMC layers were calculated. No significant differences were found between the samples from the point of view of the upper IMC layer. This was between 2.2 ± 0.2 μm, and it was continuous round-type (Figure 7A–C). 

The upper IMC layers probably remained the same since the volume fraction of the TiO_2_ decreased at the upper region of the solder joints, as was found by Nasir et al. [38]. However, the ceramic particles suppressed the Cu_6_Sn_5_ IMC layer growth significantly at the bottom. The average bottom IMC layer thicknesses were 2.72 ± 0.32 μm (SACX0307), 1.94 ± 0.17 μm (SACX0307-TiO_2_), and 2.1 ± 0.91 μm (SACX0307-nanoTiO_2_) (Figure 7D–F). The use of the ceramic particles caused a ~30% decrease in the bottom Cu_6_Sn_5_ IMC layer thickness, the thickness of the Cu_3_Sn layer did not change. The type of the bottom IMC layer was an elongated scallop-type in the case of SACX0307 and a rough scallop-type in the case of SACX0307-TiO_2_ and SACX0307-nanoTiO_2_ samples. Tang et al. [39] also found that an increase in TiO_2_ nanoparticles from 0.02 to 0.1 wt.% changes the morphology of the Cu_6_Sn_5_ IMC layer from an elongated scallop-type to rough scallop-type. A further increase in the TiO_2_ particle proportion (0.3 and 0.6 wt.%) can cause the growth of wicker-type Cu_6_Sn_5_ grains [27,39]. 

In the case of the composite solder joints, wicker-type IMC grains formed. These wicker-type Cu_6_Sn_5_ grains were several ten to several hundred micrometers in length. Tsao et al. [31] wrote about the appearance of wicker-type Cu_6_Sn_5_ IMCs in the case of adding TiO_2_ nanoparticles to Sn3.5Ag0.25Cu solder alloy. They found that wicker-type IMCs grow from the top of scallop-type IMCs, so nucleation is not required. It was explained that the growth is initiated by a local concentration increase in Cu. The whicker-type IMC elongates into the solder matrix due to the reaction of Sn with this Cu. The presence of TiO_2_ particles in the molten solder could influence the sudden growth in Cu concentration at given places, although further research into this issue is necessary.

The changes in the Cu_6_Sn_5_ IMC layer in the composite solder joints can be explained by the following: while being soldered, the dispersed TiO_2_ particles in the molten solder precipitate at the bottom of the solder joint. According to heterogeneous nucleation theory, the presence of TiO_2_ particles decreases the thermodynamic energy of Cu_6_Sn_5_ nucleation since the Cu_6_Sn_5_ prefers to nucleate on the TiO_2_ particles. The increase in the volume fraction of TiO_2_ particles means more and more nucleation sites and the increase in nucleation probability of Cu_6_Sn_5_ grain growth. This increases the nucleation rate and results in the refinement of Cu_6_Sn_5_ grain by blocking the sequential grain ripening. According to Ostwald ripening theory, the size differences between neighboring IMC grains cause the solder flux to diffuse towards the bigger grains and away from the smaller ones. This results in the further shrinkage of smaller grains and enlargement of bigger grains, so the large grains grow by absorption of atoms which have diffused from the small ones [39]. This phenomenon was also observed in our research in the composite solder joints, namely some larger Cu_6_Sn_5_ grains formed between the refined Cu_6_Sn_5_ grains (Figure 7E,F).

Furthermore, the suppression of Cu_6_Sn_5_ IMC layer growth can also be explained by the adsorption theory of TiO_2_ particles as a surface-active material. The surface energy of the Cu_6_Sn_5_ grains is minimized in the equilibrium state. An increase in the adsorption of an active element like TiO_2_ particles decreases this surface energy. According to the Gibbs free energy equation, surface energy reduction decreases the growth velocity of the Cu_6_Sn_5_ grains and results in the suppression of the whole Cu_6_Sn_5_ IMC layer growth. It also has to be noted that the presence of TiO_2_ particles in the solder matrix can act as diffusion barriers of Cu and Sn atoms, which are responsible for the further growth of the IMC layer in the solid-state of the solder joint. According to our observations, the thickness of the IMC layer was also decreased by the spalling of the IMCs into the solder bulk. The traces of the more intensive spalling are clearly visible in the composite solder joints (Figure 7E,F). This spalling/braking phenomenon of the IMC grains has also been reported by other researchers [39,40]. Tang et al. [39] showed that the IMC grains contain twice the number of weight percentages of Ti and O than the surface of the Cu_6_Sn_5_ IMC grain. This can cause the more intensive spalling/braking of the IMCs in the composite solder joints.

Some refinement of the Ag_3_Sn IMCs was observed in the SACX0307-TiO_2_ solder joints. The Ag_3_Sn particles were evenly distributed, and they were isolated with larger spacing between them (Figure 7E) than in the case of the other two sample types. In the case of the reference SACX0307 and SACX0307-nanoTiO_2_ solder joints (Figure 7D,F), Ag_3_Sn particles frequently agglomerated to the groups. The reason could be that during the solidification process, the TiO_2_ particles ensure a high nucleation density of the second phase in the eutectic colony [41]. Chung et al. [42] and Tsao et al. [31] observed the same phenomenon in the case of different composite solders joints. Interestingly, this effect was not observed in the case of nano TiO_2_ particles.

**Figure 8:**
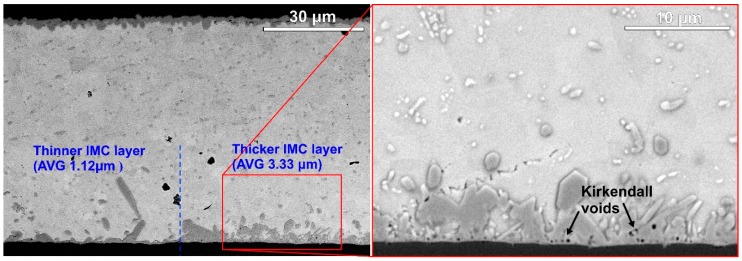
BSE-SEM micrographs of a SACX0307-nanoTiO_2_ solder joint under the thermal pad.

Although the TiO_2_ nanoparticles decreased the average bottom IMC layer thickness to 2.1 ± 0.91 μm, the deviation was very high. The reason for the high deviation was examined further, and it was found that the IMC layer thickness could differ significantly, even in one given sample. The most extreme IMC layer thickness differences were found over the thermal pad, where most of the dissipated heat leaves the power LED. Figure 8 shows a sample part of the SACX0307-nanoTiO_2_ solder joint, where the IMC layer thickness changed suddenly and considerably.

On the right-hand side of the solder joint, the IMC layer growth was suppressed effectively, the average thickness was around 1 µm. On the left-hand side of the solder joint, the IMC layer was even thicker than in the reference SACX0307 sample, the average thickness almost reached 3.5 µm. The Kirkendall voids were also more frequent at these thicker IMC layer regions. Considerable local increases in the bottom IMC layer thickness and Kirkendall voids were frequent over the thermal pad. No direct evidence was found which could explain these phenomena. It might also be related to the local concentration increase in Cu, like in the case of wicker-type IMC growth, since, in both cases, considerable IMC growth occurred. However, the role of the TiO_2_ particle size is unclear, and why it occurred only in the case of nanoparticles; further research into this issue is necessary.

The Sn grain refinement in the composite solder joints results in considerable mechanical stability improvement, as was reported by Nasir et al. [38]. However, the size and orientation of the Sn grain do not have a significant effect on the heat conduction ability of the solder joints. Nevertheless, the IMC layer acts as a continuous heat insulator layer in the solder joints between the contact pads and the solder bulk. The specific heat conductivity of the Sn and the Cu_6_Sn_5_ is 73.3 W/mK and 34.1 W/mK, respectively. It could decrease the *R_th_* and *R_the_* values of power LED assemblies soldered with SACX0307-TiO_2_. The spalling of IMCs does not change the volume fraction of the Cu_6_Sn_5_ in the solder joints. The IMCs are only more distributed in the solder joint and not concentrated on the Cu and Sn interface. More distributed Cu_6_Sn_5_ IMCs ensure heat paths with lower heat resistance.

The average IMC layer thickness also decreased in the SACX0307-nanoTiO_2_ solder joints (compared to the reference SACX0307), but a considerable and local increase in the bottom IMC layer thickness and the Kirkendall voids were frequently found over the thermal pad. The Kirkendall voids also decrease the thermal and electrical conductivity of the solder joints [43]. This could explain that the thermal properties of the SACX0307-nanoTiO_2_ solder joints were not improved compared to the reference SACX0307 solder joints. The minor refinement of the Ag_3_Sn IMCs in the SACX0307-TiO_2_ solder joints probably has no considerable effect on the heat conductivity of the solder joints, but it could mean further mechanical improvement [21]. 

## 4. Conclusions

The effect of the microstructure of solder joints on the thermal properties of power LEDs was investigated. Adding 1 wt.% submicron TiO_2_ particles decreased the thermal and electric thermal resistances of the light sources by 20% and 16%, respectively, and it slightly increased luminous efficiency. Adding 1 wt.% nano TiO_2_ particles slightly increased the thermal and electric thermal resistances of the light sources and decreased the luminous efficiency by 12% compared to the reference samples. Microstructural evaluations showed that the TiO_2_ particles were incorporated at the Sn grain boundaries and at the interface of the IMC layer and the solder bulk. The TiO_2_ particles caused refinement of the Sn grain structure of two orders of magnitude, from hundreds of micrometers to 3–5 µm. The precipitated TiO_2_ particles at the bottom of the solder joint decreased the thermodynamic energy of Cu_6_Sn_5_ nucleation since the Cu_6_Sn_5_ prefers to nucleate on the TiO_2_ particles. This refined the Cu_6_Sn_5_ grains in the composite solder joints and changed the IMC layer structure from elongated scallop-type to rough scallop-type, which means some larger Cu_6_Sn_5_ grains between the refined ones. Usually, the adsorption of TiO_2_ particles into the Cu_6_Sn_5_ grains decreased the surface energy, which suppressed the growth of Cu_6_Sn_5_ IMC layer and enhanced the spalling of Cu_6_Sn_5_ grains to the solder bulk. However, considerable local increases in the bottom IMC layer thickness and Kirkendall voids were frequently found over the thermal pad in the SACX0307-nanoTiO_2_ solder joints. The modified IMC structure of the SACX0307-TiO_2_ solder joints decreased their thermal resistance and resulted in better luminescence properties in power LED assemblies. The addition of TiO_2_ nanoparticles had similar effects on the microstructure of the solder joints, but it did not result in thermal or electrical improvements; further research is necessary. These findings are important for the further development of composite solder alloys as well as power LED light sources. 

## Figures and Tables

**Table 1:** Elemental composition of the measurement points M1–M6 in the solder joints.

EDX Measurement Point / Element [at.%]	Sn	Ag	Cu	Ti
M1	40.3	57.2	2.5	0
M2	96.8	0.4	2.6	0.2
M3	80.0	0.8	17.8	1.4
M4	43.9	0	56.1	0
M5	27.6	0	72.4	0
M6	1.1	0	98.9	0
